# *Salmonella* Hadar linked to two distinct transmission vehicles highlights challenges to enteric disease outbreak investigations

**DOI:** 10.1017/S0950268824000682

**Published:** 2024-09-05

**Authors:** Joshua M. Brandenburg, Gregory Sean Stapleton, Kelly E. Kline, Jennifer Khoury, Krystle Mallory, Kimberly D. Machesky, Stephen G. Ladd-Wilson, Ryan Scholz, Jennifer Freiman, Colin Schwensohn, Alexandra Palacios, Laura Gieraltowski, Zachary Ellison, Beth Tolar, Hattie E. Webb, Kaitlin A. Tagg, Zainab Salah, Megin Nichols

**Affiliations:** 1 Division of Foodborne, Waterborne and Environmental Diseases, Centers for Disease Control and Prevention, Atlanta, GA, USA; 2 Oak Ridge Institute for Science and Education, Oak Ridge, TN, USA; 3 Pennsylvania Department of Health, Harrisburg, PA, USA; 4 Kentucky Department for Public Health, Frankfort, KY, USA; 5 New Hampshire Division of Public Health Services, Concord, NH, USA; 6 Ohio Department of Health, Columbus, OH, USA; 7 Oregon Health Authority, Public Health Division, Portland, OR, USA; 8 Oregon Department of Agriculture, Salem, OR, USA; 9 U.S. Department of Agriculture, Office of Public Health Science, Food Safety and Inspection Service, Washington, DC, USA; 10 ASRT, Inc., Suwanee, GA, USA

**Keywords:** outbreaks, public health, *Salmonella*, zoonotic foodborne diseases

## Abstract

In 2020, an outbreak of *Salmonella* Hadar illnesses was linked to contact with non-commercial, privately owned (backyard) poultry including live chickens, turkeys, and ducks, resulting in 848 illnesses. From late 2020 to 2021, this *Salmonella* Hadar strain caused an outbreak that was linked to ground turkey consumption. Core genome multilocus sequence typing (cgMLST) analysis determined that the *Salmonella* Hadar isolates detected during the outbreak linked to backyard poultry and the outbreak linked to ground turkey were closely related genetically (within 0–16 alleles). Epidemiological and traceback investigations were unable to determine how *Salmonella* Hadar detected in backyard poultry and ground turkey were linked, despite this genetic relatedness. Enhanced molecular characterization methods, such as analysis of the pangenome of *Salmonella* isolates, might be necessary to understand the relationship between these two outbreaks. Similarly, enhanced data collection during outbreak investigations and further research could potentially aid in determining whether these transmission vehicles are truly linked by a common source and what reservoirs exist across the poultry industries that allow *Salmonella* Hadar to persist. Further work combining epidemiological data collection, more detailed traceback information, and genomic analysis tools will be important for monitoring and investigating future enteric disease outbreaks.

## 1. Introduction

Non-typhoidal *Salmonella enterica* causes over one million infections in the United States annually [1, 2]. Multistate outbreaks of *Salmonella* infections occur every year and are linked to food products or contact with animals or their environments [3]. Salmonellosis is a nationally notifiable disease in the United States [4]. When *Salmonella* is isolated by culture from infected people’s specimens, state and local public health laboratories perform whole-genome sequencing (WGS) on resulting bacterial isolates and upload the data to PulseNet, the national molecular subtyping network for enteric disease surveillance centralized at the United States Centers for Disease Control and Prevention (CDC) [5–7]. PulseNet utilizes core genome multilocus sequence typing (cgMLST) analysis to detect nationwide outbreaks of salmonellosis. CDC, along with federal, state, and local public health partners, will initiate outbreak investigations if *Salmonella* isolates are temporally clustered and cgMLST analysis indicates a high degree of genetic relatedness. Genetically related isolates are more likely to share a common transmission source [5]. Public health officials conduct interviews of ill people to identify possible sources of infection and to direct further laboratory testing and traceback of contaminated foods or animal reservoirs. Investigation of outbreaks of genetically related isolates might identify a discrete source of contamination to target interventions for preventing illnesses, but investigations might also fail to identify a source or might reveal that a strain is widely disseminated across a specific industry [8, 9].

Non-commercial, privately owned (also referred to as ‘backyard’) poultry, such as chickens, turkeys, and ducks, are an increasingly common source of zoonotic transmission of *Salmonella* because of their growing popularity in the United States [10]. Poultry can harbour *Salmonella* in their gastrointestinal tract that can be intermittently shed in excreta and transmitted to humans, even while the animal appears healthy. Backyard poultry contact is commonly associated with sporadic human *Salmonella* illness, and multistate outbreaks linked to backyard poultry occur annually, coinciding with the increased sale and distribution of backyard poultry across state lines in the spring every year. There are approximately 20 mail-order hatcheries throughout the United States that contribute most of the backyard poultry to US consumers, either directly to consumers from the hatchery or indirectly to consumers through hatcheries partnering with one another and sharing distribution or by supplying agricultural feedstores [11, 12]. Previous studies have described poultry sourcing and distribution practices among mail-order hatcheries [13]. Investigations of backyard poultry-associated *Salmonella* outbreaks have identified specific sources of contamination along the distribution chain [14], but these outbreak strains might also be widely disseminated among backyard poultry hatcheries and retailers [12]. This growing problem necessitates public health intervention through owner education as well as industry-level pathogen mitigation efforts [13].

Consumption of contaminated poultry products is a major contributor to the overall burden of *Salmonella* infections and can result in *Salmonella* illness outbreaks [15]. Historically, outbreaks of foodborne *Salmonella* Hadar infections were most commonly associated with retail turkey products [16]. Turkeys raised to be slaughtered and processed for food are produced through systems that are generally distinct from those that provide animals to the backyard poultry market. Poultry raised for the commercial food industry are usually not sold live to the public. Individuals wishing to obtain backyard poultry may buy through agricultural feedstores that are supplied by hatcheries, mail orders direct from hatcheries, or private farms or flea markets [13]. Therefore, multistate *Salmonella* Hadar outbreaks where a closely related genetically outbreak strain has been attributed to both backyard poultry and poultry food products have not been previously reported, to our knowledge. However, implementation of WGS has improved our ability to detect *Salmonella* in different products.

In 2020, backyard poultry were implicated as the cause of a multistate outbreak of *Salmonella* Hadar infections. Later that year and in 2021, CDC, along with federal and state partners, investigated another multistate outbreak of *Salmonella* Hadar and identified ground turkey as the source of illness [17]. *Salmonella* Hadar isolates obtained from both outbreaks were highly related based on cgMLST analysis. This study compares the investigations and findings of each outbreak and examines explanations provided by epidemiological and advanced genomic analyses underlying the phenomenon of two outbreaks with exposures to distinct vehicles resulting from a closely related genetically *Salmonella* Hadar strain.

## 2. Methods

The reported outbreak investigation activities were reviewed by the CDC and were conducted in accordance with the applicable federal law, and US Department of Agriculture Food Safety and Inspection Service (FSIS) policy.^§^

### 2.1. Backyard poultry-associated outbreak

In April 2020, PulseNet notified CDC epidemiologists of 15 ill people from 11 states infected with *Salmonella* Hadar that was genetically related within 0–7 allele differences by cgMLST analysis. Preliminary data available on patient exposures through routine state or local health department interviews indicated nine of ten ill people with available information reported contact with backyard poultry. State and local public health officials continued to collect and share patient exposures (including foods eaten and animals contacted, among other general exposures) identified through routine state or local health department interviews throughout the duration of the investigation. Public health officials conducted additional interviews with patients whenever possible with a supplemental standardized questionnaire examining types of poultry exposure and poultry purchase locations such as feedstores, local farms, and agricultural co-ops. Ill people were asked about their poultry purchasing since 1 January 2020, thus allowing investigators to better identify traceable records from purchase location to source hatchery. During interviews, ill people were asked whether they were willing to have their backyard flocks sampled for *Salmonella.* Questionnaire responses were collected in CDC’s Epi Info™ Web Survey and aggregated using the System for Enteric Disease Response, Investigation, and Coordination (SEDRIC) [18, 19]. A case was ultimately defined as *Salmonella* Hadar infection yielding an isolate, related within 0–15 allele differences based on cgMLST, from a patient with illness onset dates from 26 February 2020 through 11 November 2020 [9, 20, 21]. Patient response data were analysed using Statistical Analysis System (SAS) software, version 9.4 (Cary, NC, USA). All clinical isolates have been deposited to the National Center for Biotechnology Information (NCBI) BioProject PRJNA230403.

State and local public health and agricultural officials in Kentucky, New Hampshire, and Oregon conducted the sampling of backyard poultry and their environments at ill people’s homes using standard procedures [22]. These samples were processed by their respective public health laboratories utilizing standardized aerobic culture methods [23] and PulseNet WGS protocols [20]. WGS data were uploaded to the PulseNet national database and compared to outbreak patient sequences.

CDC epidemiologists utilized information from patient interviews to identify any backyard poultry hatcheries or suppliers that could have been a common source of backyard poultry resulting in the transmission of *Salmonella* Hadar in this outbreak. Some ill people reported how and where they acquired their poultry, many of whom had purchased from agriculture feedstores. Some feedstore locations were part of corporations; the CDC shared purchase information for purchases since 1 January 2020 with feedstore corporations (>100 store locations) to identify the hatcheries that supplied poultry to their stores. Employees of independent feedstores, farms, agriculture co-ops, and small feedstore corporations (<100 store locations) where ill people had purchased poultry were interviewed with a standardized questionnaire regarding poultry breeds and species sold and source hatcheries based on ill people’s reported purchase dates.

### 2.2. Ground turkey-associated outbreak

In February 2021, PulseNet notified CDC epidemiologists of 17 cases of *Salmonella* Hadar infection with specimen collection dates since 1 January 2021 that were related within 10 allele differences by cgMLST analysis. These isolates were also genetically related to the 2020 backyard poultry-associated outbreak. Because of genetic similarities between patient isolates, ground turkey isolates, and isolates from the 2020 backyard poultry-associated outbreak in the PulseNet database, state and local health officials collected information on the types of poultry products consumed in the seven days before illness onset, including brand and packaging information and location of purchase, as well as exposures to backyard poultry. Ill people were asked whether they had food products available for *Salmonella* testing. Questionnaire responses were aggregated using SEDRIC. A case was defined as *Salmonella* Hadar infection yielding an isolate, related within 0–8 allele differences based on cgMLST, from a patient with illness onset dates occurring from 28 December 2020 to 22 April 2021 [9, 20, 24].

During the course of the outbreak investigation, the FSIS tested one unopened ground turkey sample collected from a patient’s home. This sample was processed utilizing standardized FSIS *Salmonella* culture and WGS protocols [25, 26]. The FSIS carries out routine testing of turkey products and caecal samples for enteric pathogens such as *Salmonella* as part of ongoing surveillance throughout the year either via standard food safety monitoring dictated by federal directive [27] or as part of the National Antimicrobial Resistance Monitoring System (NARMS) [28]. The US Food and Drug Administration (FDA) oversees *Salmonella* testing of ground turkey purchased from retail establishments through the NARMS programme [29]. Sampling, culture methods, and WGS of *Salmonella* isolates performed by the FSIS and FDA follow standard protocols described elsewhere [25, 26, 30]. WGS data of these isolates are routinely uploaded to PulseNet.

The FSIS staff obtained information for *Salmonella*-positive retail ground turkey samples to determine where the products were processed. They also worked with public health partners to obtain patient product purchase records from information reported in patient interviews (i.e. retail store shopper card numbers) to determine whether there was a common processing establishment or brand associated with patient illness.

## 3. Results

### 3.1. Backyard poultry-associated outbreak

**Figure 1.**
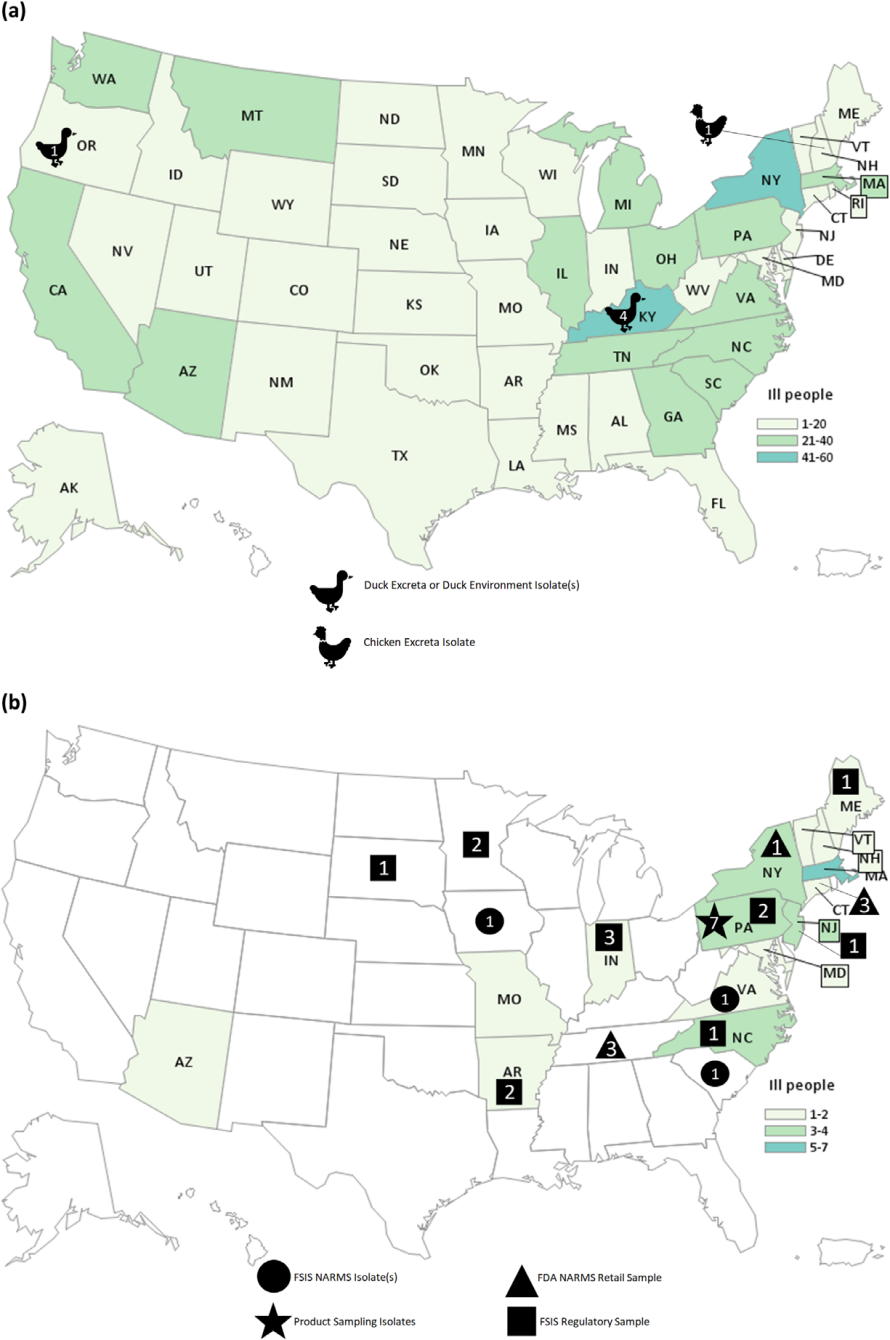
People infected with the strain of *Salmonella* Hadar by state of residence, identified as part of the backyard-poultry-associated outbreak (a) and ground-turkey-associated outbreak (b). Icons and the number within correspond to the number of isolates from that sample type.

**Figure 2.**
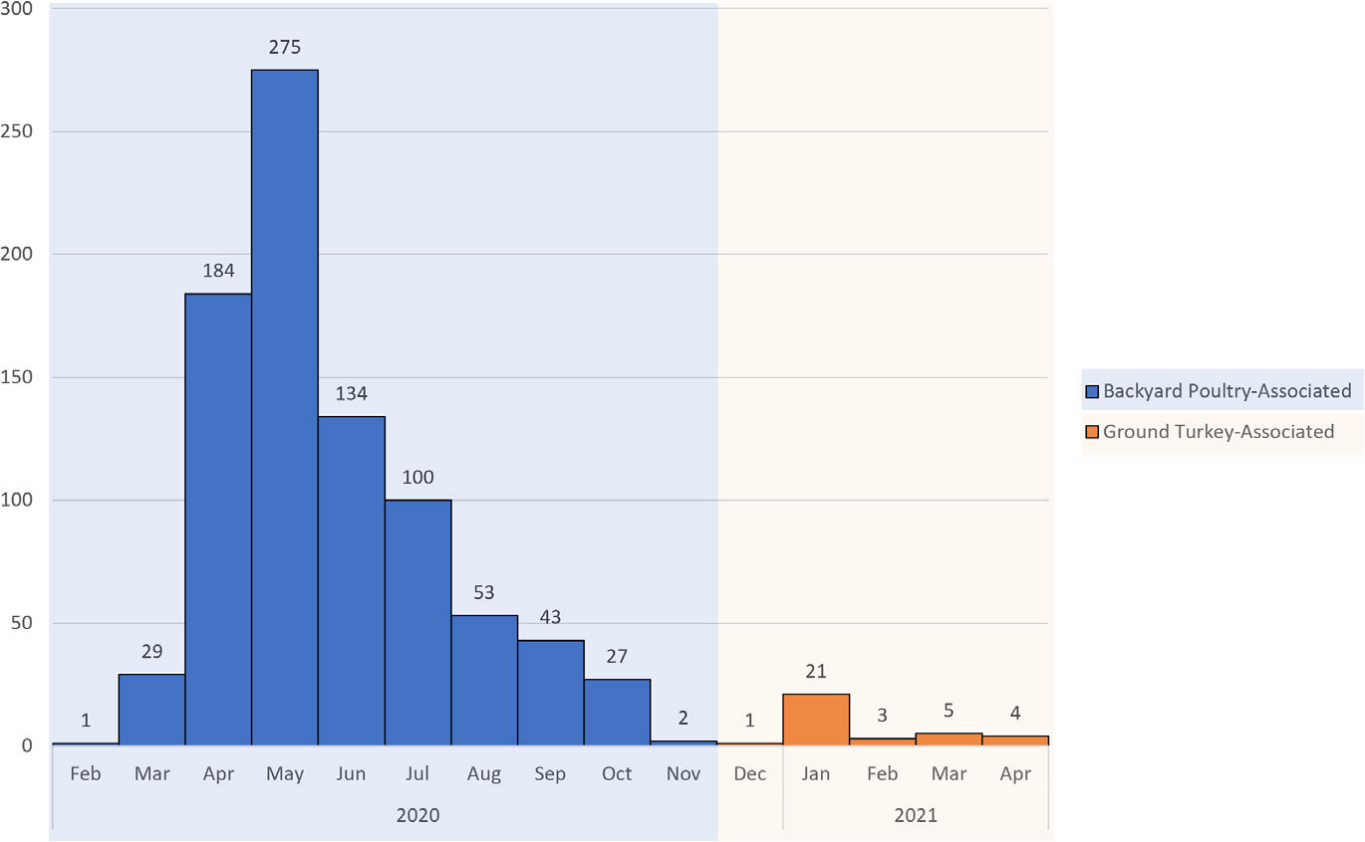
Epidemic curve of reported illnesses by onset date. People infected with the backyard poultry-associated outbreak strain of *Salmonella* Hadar (*n* = 848) and people infected with the ground turkey-associated outbreak strain of *Salmonella* Hadar (*n* = 34) by date of illness onset, United States, 2020–2021.

The investigation identified 848 people infected by the outbreak strain in 49 states (Figure 1a). Illness onset dates ranged from 26 February 2020 to 11 November 2020 (Figure 2). Ages ranged from <1 to 95 years with a median of 36 years, and 216 of 840 (26%) were children under the age of 5 years; 480 of 811 (59%) were female. Of ill people with available information, 186 of 542 (34%) were hospitalized, and there were no reported deaths. Of 476 ill people with animal exposure information available from either routine or supplemental interviews, 346 (73%) reported contact with backyard poultry. Among 159 ill people who provided information about the types of poultry they had contact with, most reported contact with chickens (70%, n = 112) or ducks (43%, n = 69). Ill people also reported contact with other poultry including turkeys (5%, n = 8), geese (3%, n = 5), or guineas (3%, n = 5). These patients primarily described the poultry they contacted as ‘baby’ poultry (76%, n = 121), while some had contact with ‘adult’ poultry (30%, n = 49). Ill people were also queried about the breeds or types of chickens or ducks that they contacted; 109 ill people provided the breed or type of chicken contacted, and 33 ill people provided the breed or type of duck contacted. A total of 28 different breeds of chickens and 15 breeds of ducks were reported. Among 438 ill people with routine interview data shared, 25 ill people reported turkey consumption of various types (i.e. ground, deli/sliced), 8 of which also had contact with backyard poultry prior to their illness onset.

Testing of poultry and their environment yielded six *Salmonella* Hadar isolates: four isolates were collected from three duck cloacae and their environment at a patient’s home in Kentucky, one was obtained from another duck pen area at an ill patient’s home in Oregon, and one was obtained from a chicken’s excreta at a patient’s home in New Hampshire. All six isolates were highly related to each other and the corresponding patient isolates within 0–4 allele differences. No poultry feed samples were tested.

**Figure 3.**
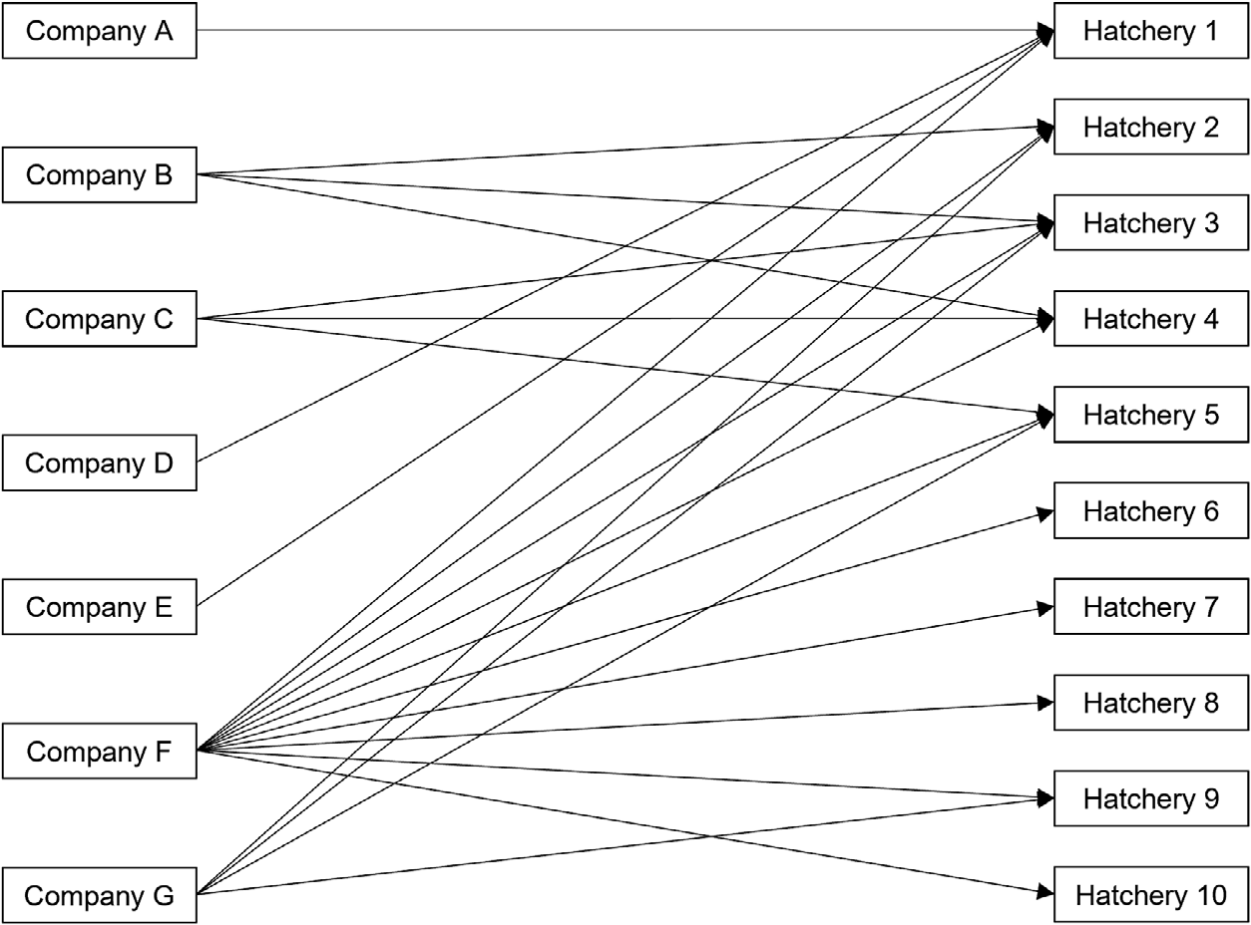
Traceback diagram depicting 10 hatchery sources among 7 of 48 (15%) companies with traceable poultry purchase locations in the backyard-poultry-associated outbreak.

Among 346 ill people with backyard poultry contact, 210 (61%) reported purchasing poultry since 1 January 2020. A total of 210 ill people reported 223 distinct purchases from at least 48 companies, including mail-order hatcheries, corporate and independent farms, or feedstores, from 155 unique locations. Eight questionnaires administered to storefronts across five independently owned and operated companies were returned detailing where they sourced poultry. Additionally, source hatcheries were identifiable for 26 store locations included as part of two large corporations. In total, 34 (22%) purchase locations belonging to seven companies provided source hatchery information. These store locations were traced to 10 different hatcheries located in eight states (Figure 3). For hatcheries identified in traceback, information could not be obtained regarding the sources of poultry among these hatcheries or whether these hatcheries shared any common suppliers.

### 3.2. Ground turkey-associated outbreak

This investigation identified 34 people ill with the outbreak strain from 15 states (Figure 1b). Illness onset dates occurred from 28 December 2020 to 22 April 2021 (Figure 2). Ages ranged from <1 to 92 years, with a median of 49 years, and 21 (62%) ill people were female. A total of 4 (18%) of 22 patients with available information were hospitalized, and no deaths were reported. Thirteen ill people responded to requests for an interview with the questionnaire. Of these 13 ill people who were asked specifically about turkey exposures, eight (62%) reported eating ground turkey within 7 days of becoming ill. This was significantly higher than the 13% of healthy people who reported eating ground turkey the week prior to the interview in the 2018–2019 FoodNet Population Survey (p < 0.001) [31]. An additional two people reported eating turkey products other than ground turkey within 7 days of becoming ill. Ill people reported purchasing seven different brands of turkey products. No ill people reported owning or contacting backyard poultry directly. One patient reported eating chicken and duck eggs provided by their neighbour.

A total of 29 isolates of the outbreak strain were obtained from turkey samples from 14 slaughter or processing establishments: 12 isolates detected through FSIS regulatory sampling of ground turkey at production facilities, three isolates obtained through FSIS NARMS sampling of turkey caeca, seven isolates identified through NARMS surveillance efforts by the FDA and state partners of retail ground turkey products, and seven isolates from an unopened package of ground turkey at an ill patient’s home in Pennsylvania. Six of the seven isolates from the product at the Pennsylvania home were indistinguishable (0 allele differences) from the isolate collected from the patient. Isolates from ill people, turkey caecal contents, and ground turkey products were related within 0–8 allele differences by cgMLST. The ground turkey sampled from the ill patient’s home also yielded six isolates of *Salmonella* serotype I 3,10:e,h:-, which was not isolated from any ill people or genetically related to the outbreak strain. One isolate of genetically related *Salmonella* Hadar from a chicken product was reported through FSIS regulatory sampling. This chicken product sample was obtained from an establishment that processes both chicken and turkey products.

FSIS conducted the traceback of ground turkey purchases for six ill people from four states. No single retail store or processing establishment could be linked to all ill people. Multiple suppliers were identified during traceback; two establishments (‘Establishments X and Y’) were the sole suppliers of ground turkey purchased by two ill people each. Two ill people (one from Maryland and one from Maine) ate ground turkey product that was traced back to Establishment X; two ill people from Pennsylvania ate ground turkey that was traced back to Establishment Y; and two ill people (one from Pennsylvania who allowed testing of ground turkey remaining in their home and one from Connecticut) ate ground turkey traced back to multiple suppliers, including both Establishments X and Y. Establishments X and Y were among 14 establishments located in 11 states that had turkey isolates included in the investigation.

**Figure 4.**
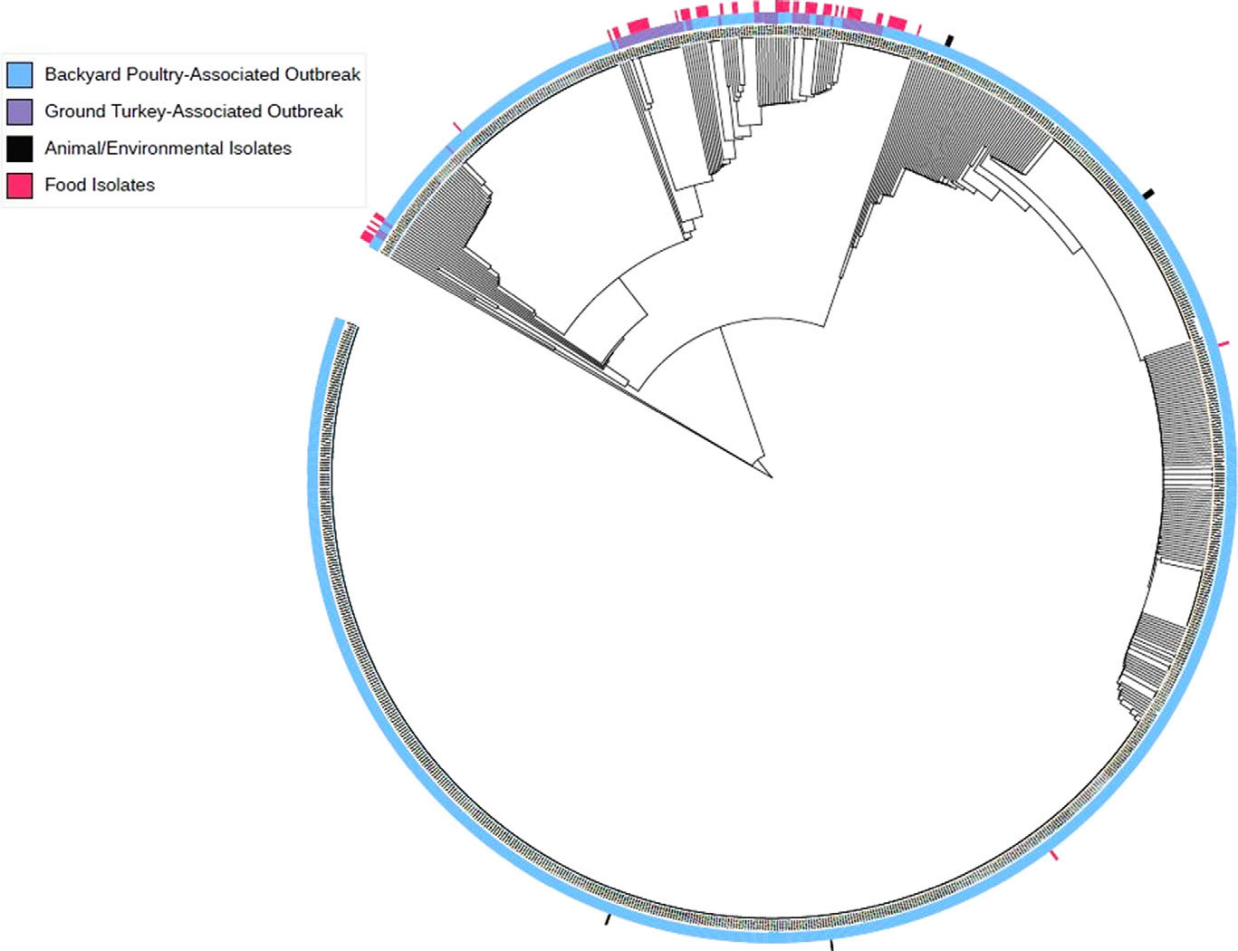
cgMLST analysis of 950 *Salmonella* Hadar isolates related by 0–16 allele differences identified during the backyard poultry-associated outbreak and ground turkey-associated outbreak from human, food, animal, or environmental sources. The inner ring (black colour) of this diagram is a phylogenetic tree demonstrating relatedness of the 950 isolates. The middle ring (blue or purple colour) designates which outbreak investigation each isolate belongs to. The outermost ring designates whether isolates were obtained from food products (pink colour) or backyard poultry or their environment (black colour); isolates without this label are clinical isolates.

Isolates from ill people included in the ground turkey-associated outbreak were closely genetically related within 0–16 alleles by cgMLST to isolates included in the backyard poultry-associated outbreak (Figure 4). No backyard poultry were sampled during the ground-turkey-associated outbreak because no ill people reported backyard poultry contact or ownership.

## 4. Discussion

We report two multistate outbreaks linked to distinct vehicles but caused by *Salmonella* Hadar that was closely genetically related as determined by cgMLST (within 0–16 alleles). The emergence of this strain in 2020, the high number of illnesses that resulted, the persistence of transmission, and the dissemination in backyard poultry and food poultry industries are of public health concern. Backyard poultry-associated *Salmonella* Hadar illnesses contributed to an overall 617% (95% CI: 382–987%) increase in *Salmonella* Hadar in 2020 compared to 2017–2019 [32]. Additionally, *Salmonella* Hadar is one of the most common serotypes isolated from food-producing turkeys and derived products in North America [33–35]. Turkey products have contributed to both single and multistate outbreaks of *Salmonella* Hadar in the United States [16], but it has not been previously established that these outbreaks are genetically, epidemiologically, or otherwise related to *Salmonella* Hadar strains transmitted to people from backyard poultry.

The two outbreaks reported here were investigated as two distinct events, and the epidemiological, laboratory, and traceback evidence collected during these investigations have yet to explain how these outbreaks, linked to distinct vehicles, resulted from a closely genetically related *Salmonella* Hadar strain (within 0–16 alleles by cgMLST). During the backyard poultry-associated outbreak, ill people might have been asked about food exposures through routine state or local health department interviews, but these questions are not standardized across jurisdictions; exposure to turkey products was reported by ill people but was infrequent, with a small number of ill people reporting backyard poultry exposure and turkey consumption. Of note, not all ill people in the backyard poultry-associated outbreak were asked about turkey food product exposure, and reporting might have been subject strictly to patient recall when asked about general food exposures in the week prior to illness onset. This could have artificially reduced the number of ill people in this outbreak reporting ground turkey exposure. Furthermore, routine sampling of turkey by the FSIS, the FDA, and state and local public health officials was ongoing throughout the backyard poultry-associated outbreak [27–29]. The outbreak strain was detected in ground turkey during the backyard poultry-associated outbreak investigation, but because of the increased number of ill people reporting backyard poultry contact during that time, additional follow-up of ground turkey consumed by patients was not conducted as part of the backyard poultry-associated outbreak investigation. Systematically questioning patients about food poultry exposures during this investigation could have revealed that some people were becoming ill as a result of ground turkey at the same time that people were known to be exposed to *Salmonella* Hadar via contact with backyard poultry, and this could have identified additional measures to prevent illnesses during this outbreak. During the ground turkey-associated outbreak, ill people were specifically asked about exposure to backyard poultry, and none reported direct contact or ownership.

In both outbreaks, some ill people could not be interviewed, and no exposure information was available from them, as is typical for enteric disease outbreak investigations. Therefore, it is possible that ill people in either outbreak were exposed to the outbreak strain by a different vehicle. These *Salmonella* Hadar outbreaks illustrate the importance of collecting detailed epidemiological evidence to characterize food and animal exposures. When further outbreaks of this *Salmonella* Hadar strain occurred after 2021, investigators questioned ill people in detail about their exposure to food turkey products and backyard poultry, and this has aided in determining which ill people have been exposed to contaminated foods and which by animal contact.

cgMLST analysis demonstrates that food, animal, and clinical isolates from both outbreaks were closely genetically related (within 0–16 alleles). In 2019, WGS became the standard molecular subtyping approach for foodborne disease surveillance across PulseNet participating public health laboratories; this replaced the previous method of pulsed-field gel electrophoresis (PFGE) and introduced substantially higher precision when identifying ill people during outbreak investigations [5, 36]. This was particularly useful in distinguishing isolates of clonal *Salmonella* serotypes that demonstrate minimal genetic variation over time and were indistinguishable by PFGE [5]. *Salmonella* Hadar demonstrates such clonality; of 3,047 isolates of *Salmonella* Hadar available in the PulseNet database as of July 2023, 2,143 (70%) are related within 0–26 alleles by cgMLST [37]. cgMLST compares genes identified in >97% of the strains of a given bacterial species, which, in *Salmonella*, consists of 3,002 loci [24, 38]; however, this does not examine the accessory genome of isolates, which is a collection of highly variable genes that might be shared between bacteria via horizontal transfer as plasmids, transposable elements, or other mobile genetic material [39]. Different methods might be employed to analyse WGS data that provide varying degrees of granularity in evaluating the genetic relatedness between strains. In future, analysis of the complete *Salmonella* Hadar pangenome might allow distinction between source exposures in ill people infected with this strain, and at the time of writing, the authors are investigating the utility and limitations of such an analytic approach for describing *Salmonella* Hadar.

In addition to epidemiological and laboratory evidence, traceback investigations conducted during both outbreaks were not able to explain how backyard poultry could be linked to or transmit *Salmonella* Hadar that some people later acquired from exposure to or consumption of contaminated ground turkey. Ill people in the backyard poultry-associated outbreak primarily reported contact with chickens and ducks, and live turkey contact was reported infrequently. It is unknown how frequently poultry sold for backyard keeping overlap during their life cycle with those raised and processed for commercial food production. In some instances, commercial poultry egg suppliers do supply hatching eggs or live young birds to backyard poultry hatcheries that subsequently supply agricultural feedstores [14]. However, further information needs to be collected from industry partners to fully understand whether there is a plausible connection in the poultry supply chain linking commercial food producers and backyard poultry hatcheries. One hypothesis that might explain the finding of the strain in different sectors of the poultry industry is that backyard poultry and commercially produced turkeys associated with each outbreak received the same feed that was contaminated with the implicated *Salmonella* Hadar strain. Contaminated animal feed is a documented source of *Salmonella* outbreaks in people [40]. Patient interviews did not identify a common feed administered between backyard poultry owners, nor were feed samples tested during the investigation. Additionally, while traceback of ground turkey product samples and ground turkey purchased by ill people identified processing establishments for some products, the investigation did not identify farms at which turkeys were raised before processing, thus precluding on-farm follow-up to examine potential sources of *Salmonella* Hadar, such as feed, during the outbreak. *Salmonella* Hadar has historically been one of the most common serotypes isolated from poultry feed in European studies [41, 42], but *Salmonella* is now reported in less than 0.5% of samples taken from poultry feed in the European Union [43]. The FDA Center for Veterinary Medicine monitors the presence of *Salmonella* in livestock and poultry feeds and has reported a declining prevalence of *Salmonella* over time, though *Salmonella* prevalence in feed from the United States is reportedly higher compared to the prevalence in Europe [44]. These efforts, as well as other surveillance studies, have detected *Salmonella* Hadar in poultry feed infrequently [44, 45], and some have not detected *Salmonella* Hadar et al. [46]. Ultimately, although feed is a potential commonality between backyard poultry and food production industries, there is not sufficient evidence to determine whether it was a source of *Salmonella* Hadar in these outbreaks. In the event of future outbreaks of *Salmonella* Hadar, investigators should consider testing feed samples for *Salmonella* contamination as a means of examining this hypothesis further.

Since these outbreak investigations, *Salmonella* Hadar has continued to cause illnesses in people, and additional multistate outbreak investigations have sought to characterize how these illnesses might have occurred [47]. Public health officials in the United States are continuing to identify, describe, and track strains of enteric bacteria like *Salmonella* Hadar that persistently cause illnesses over time despite investigation and prevention efforts [48]. These strains can be detected over wide geographical areas, potentially among large populations of animals or in environmental niches, and therefore, the approach to respond to and mitigate further transmission of these strains to people requires actions unique from those utilized in acute outbreaks where there is a discrete source of contamination to target interventions [48]. While focal investigations of highly related isolates remain critical to understanding the sources of these strains, for persisting strains, it is important to leverage collaboration among governmental agencies, food and animal industries, and academia to further describe where and how these strains persist – including identifying what reservoirs could be contributing to their spread and implementing strategies to reduce spread when possible. Complete elimination of these widespread persisting strains is challenging and requires time, sufficient resources, and active engagement across sectors.

This report highlights limitations to the standard epidemiological, laboratory, and traceback methods used by public health agencies to investigate *Salmonella* strains which might be widely disseminated and result in outbreaks linked to distinct transmission vehicles. Advances in genetic characterization of enteric pathogens like *Salmonella* have considerably enhanced the ability of disease investigators to respond quickly and effectively to outbreaks. However, in a complex and ever-changing globalized food system that is complicated by direct interaction with animals, new approaches and advanced technology are needed to mitigate novel threats and identify circumstances in which individual strains of enteric pathogens could be spread by different vehicles at once. This *Salmonella* Hadar strain has continued to be associated with [50] backyard poultry and ground turkey [49, 50], and public health officials have bolstered efforts to collect robust epidemiological information and are actively utilizing advanced molecular characterization techniques to learn more about this strain.

## Data Availability

The data that support the findings of this study are available from the authors upon reasonable request. All clinical isolates have been deposited to the NCBI BioProject PRJNA230403.

## Footnotes

§ See, for example, 45 C.F.R. part 46, 21 C.F.R. part 56; 42 U.S.C. §241(d); 5 U.S.C. §552a; 44 U.S.C. §3,501 et seq., 21 U.S.C. 451, et seq.
